# Continuous microfluidic synthesis of zirconium-based UiO-67 using a coiled flow invertor reactor

**DOI:** 10.1016/j.mex.2021.101246

**Published:** 2021-01-23

**Authors:** Tom Bailey, Merwyn Pinto, Nicole Hondow, Ke-Jun Wu

**Affiliations:** School of Chemical and Process Engineering, The University of Leeds, United Kingdom

**Keywords:** Metal-organic frameworks (MOFs), Continuous synthesis, Coiled flow inverter reactor (CFIR), BPDC, biphenyl-4,4-dicarboxylic acid, CFIR, coiled flow inverter reactor, DMF, dimethylformamide, MOFs, Metal organic frameworks, PXRD, powder X-Ray Diffraction, RTD, residence time distribution, SBU, secondary building unit, SEM, scanning electron microscopy, UiO, universitetet i Oslo

## Abstract

Metal-organic frameworks (MOFs), particularly Zirconium based, have a wide variety of potential applications, such as catalysis and separation. However, these are held back by traditionally only being synthesised in long batch reactions, which causes the process to be expensive and limit the amount of reaction control available, leading to potential batch to batch variation in the products, such as particle size distributions. Microfluidics allows for batch reactions to be performed with enhanced mass/heat transfer, with the coiled flow inverter reactor (CFIR) setup narrowing the residence time distribution, which is key in controlling the particle size and crystallinity. In this work, a Zirconium based MOF, UiO-67, has been synthesised continuously using a microfluidic CFIR, which has allowed for the product to be formed in 30 min, a fraction of the traditional batch heating time of 24 h. The microfluidicially synthesised UiO-67 is also smaller product with a narrower particle size distribution (≈200 nm to ≈400 nm) than its batch counterpart (~500 nm to over 3 µm).

## Graphical abstract

**Figure:**
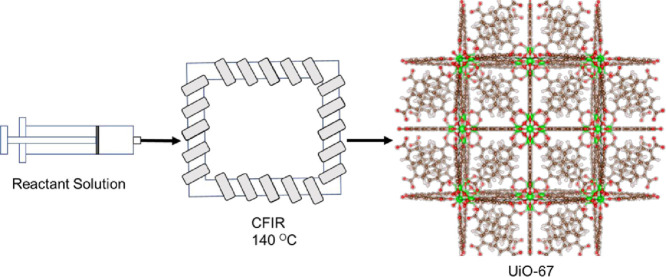

Forming UiO-67 through a continuous process using a CFIR.

Specifications table

**Table:** 

Subject Area:	Chemical Engineering
More specific subject area:	*Microfluidic Synthesis*
Method name:	*Continuous Microfluidic Synthesis of Zirconium-based UiO-67 using a coiled flow invertor reactor*
Name and reference of original method:	*Synthesis of UiO-67,* *Schaate A, Roy P, Godt A, Lippke J, Waltz F, Wiebcke M, Behrens P. Modulated synthesis of Zr‐based metal–organic frameworks: from nano to single crystals. Chemistry–A European Journal. 2011 Jun 6;17(24):6643-51.*
Resource availability:	

## Background

**Fig. 1:**
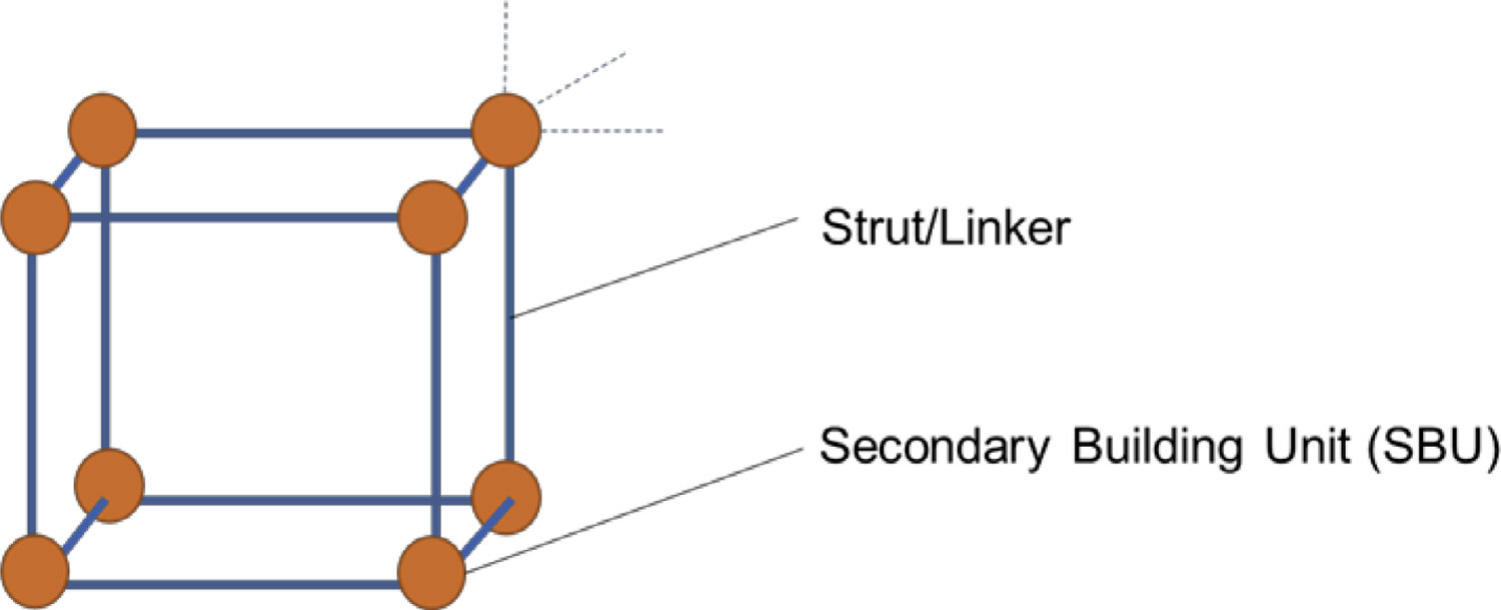
Representation of a MOF structure, with SBUs and linker units highlighted.

Metal-organic frameworks (MOFs) are a class of porous coordination polymer, discovered in 1990 with the appearance of a [N(CH_3_)_4_][CuZn(CN)_4_] cubic structure [1]. These materials are composed of metal containing secondary building units (SBUs) and organic linker units, with a general representation in Fig. 1. Due to being made up of these combinations, there is almost an unlimited number of structures theoretically available, with MOFs also prone to modification after synthesis. These MOFs can be used in a wide variety of applications, such as gas capture/separation, catalysis and drug delivery [2,3].

**Fig. 2:**
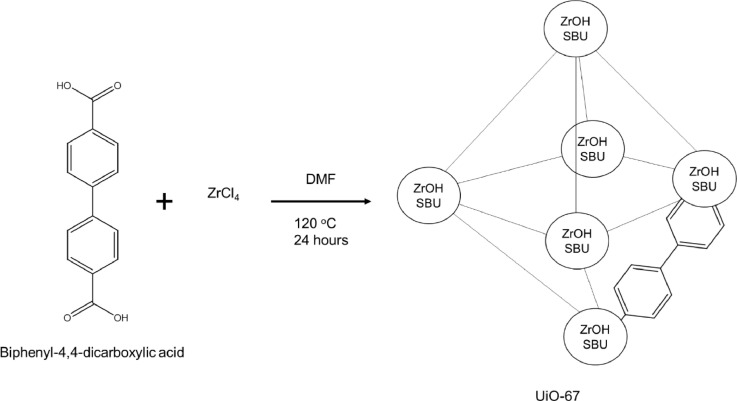
Basic reaction scheme for the synthesis of UiO-67.

Zirconium based MOFs, such as the University of Oslo (UiO) type MOFs, are of interest due to their relatively high thermal and acid/base stabilities, comparatively to non-Zr(IV) carboxylate containing MOFs [4]. They have previously been used in a range of applications, such as hydrogenation catalysis [5], adsorption of illegal organic dyes [6], modified for gas storage and many others [7]. UiO-67 was chosen for this work due to a lack of work on its microfluidic synthesis being present, when compared to the other main member of this group, UiO-66 [8,9]. UiO-67 is a larger analogue of the UiO group, so will have a higher surface area and be more efficient in absorption than UiO-66. A typical synthetic protocol for UiO-67, is represented in the Fig. 2. An issue with these MOFs however, is that their production is traditionally through the use of a lengthy batch process, with *Schaate et al* reporting a 24 h reaction time, followed by time taken to wash and dry the product [10]. Another issue with batch processes is the lack of reaction control present, which can lead to wider particle size distributions. Previous work by Zhang et al has shown that the particle size distribution of UiO-67 directly affects its absorption abilities, with a narrower distribution enhancing the absorption of organic dyes, so control over this is important [11]. A way to speed up this synthesis process and allow for further reaction control could be using microfluidic reactors.

Microfluidic synthesis occurs within micrometre sized channels, causing higher levels of mixing and heat transfer due to the higher surface area to volume ratio [12]. This higher mixing efficiency allows for greater control over the reaction, which means greater control of nanoparticle size for example [13]. Microfluidic reactors have been used to synthesis a variety of materials, such as: metal nano-rods [14], quantum dots and more recently MOFs [8,^9^,15]. A key development in the microfluidic synthesis of MOFs was published by Faustini et al, in which several MOF structures, including UiO-66, were successfully synthesised through the use of two-phase droplet synthesis, with reactant/DMF droplets being carried through a reactor by silicon oil [8]. This showed that crystalline MOFs could be formed in lower reaction times when a microfluidic system was used, but showed the system could be still improved. Mainly, the use of two phases will increase the costs of production, with large amounts of silicon oil (in a 5:1 ratio with the reactant mixture) will be used and will be largely unrecoverable for synthesis of UiO-66 at 140 °C. To simplify these systems, while still ensuring the high level of micro-mixing present in droplet reactors would be a key challenge. Following this work, Tai et al were successful in synthesising UiO-66 in a single phase microfluidic reaction, where it was found that by varying the residence time, a certain level of control could be achieved over the particle size [9]. Higher flowrates/shorter residence times results in smaller UiO-66 particles, so ensuring this residence time distribution is narrow would be key for ensuring the particle size distribution would also be narrow. Further work has been performed on other MOF groups, such as ZIF-8, which was synthesised in a microfluidic process by Kolymykov et al, while ensuring size control also through variation of the residence time [16]. They were successful in generally controlling the size through the variation of reaction conditions, but their relative particle size distributions were still high, with enhanced mixing for this system potentially being a solution. So, by adapting previous work of microfluidic based MOF synthesis, and combining it with the batch method used for the synthesis of UiO-67, it should be possible to form UiO-67 in a continuous method, while using enhanced mixing from reactor design to narrow the particle size distributions. This reactor design could then be used with future materials that would also benefit from consistent particle size.

While heat and mass transfer are improved in microfluidic systems, mixing is limited to molecular diffusion, due to the laminar flow of the system. Laminar flow also has a relatively large residence time distribution (RTD), as fluid near the channel walls will travel slower due to friction forces. To improve the mixing present in microtubular systems, the tubing can be arranged in specific patterns which may influence the mixing. One example of this is through the use of coiled flow inverter reactors (CFIRs), which arrange the tubing in helical patterns, while also including 90^o^ inversions in the flow, which enhance the mixing through secondary flow patterns appearing in the system [17]. These secondary flow patterns are called Dean Vortices and they enhance the radial mixing in the system, narrowing the RTD [18]. The 90^o^ turns cause the direction of the centrifugal force to change across the fluid, which flattens the laminar flow pattern and eliminates stagnant mixing zones in the system, which further reduces the RTD. Work performed by Wu and Torrente-Murciano found that size distribution of silver nanoparticles synthesized in this system were narrower than their straight helical counterparts, showing the enhanced mixing and lower RTDs [17]. Using this CFIR system should give MOF particles with narrower particle size distributions in a continuous method, which would be ideal for future applications where particle size will be important (e.g. absorption).

This paper describes the construction and application of a continuous CFIR microfluidic reactor for the production of UiO-67, which is a relatively small system that can be placed on a laboratory benchtop. Success in forming UiO-67 in a relatively short time, due to enhanced mixing and heat transfer in the system, highlights the potential use of this system for the formation of other MOFs in quick and continuous processes, with the reaction times and temperatures easily variable. The produced UiO-67 also showed a narrower particle size distribution, due to the enhanced mixing from the CFIR.

## Overview of the method

**Fig. 3:**
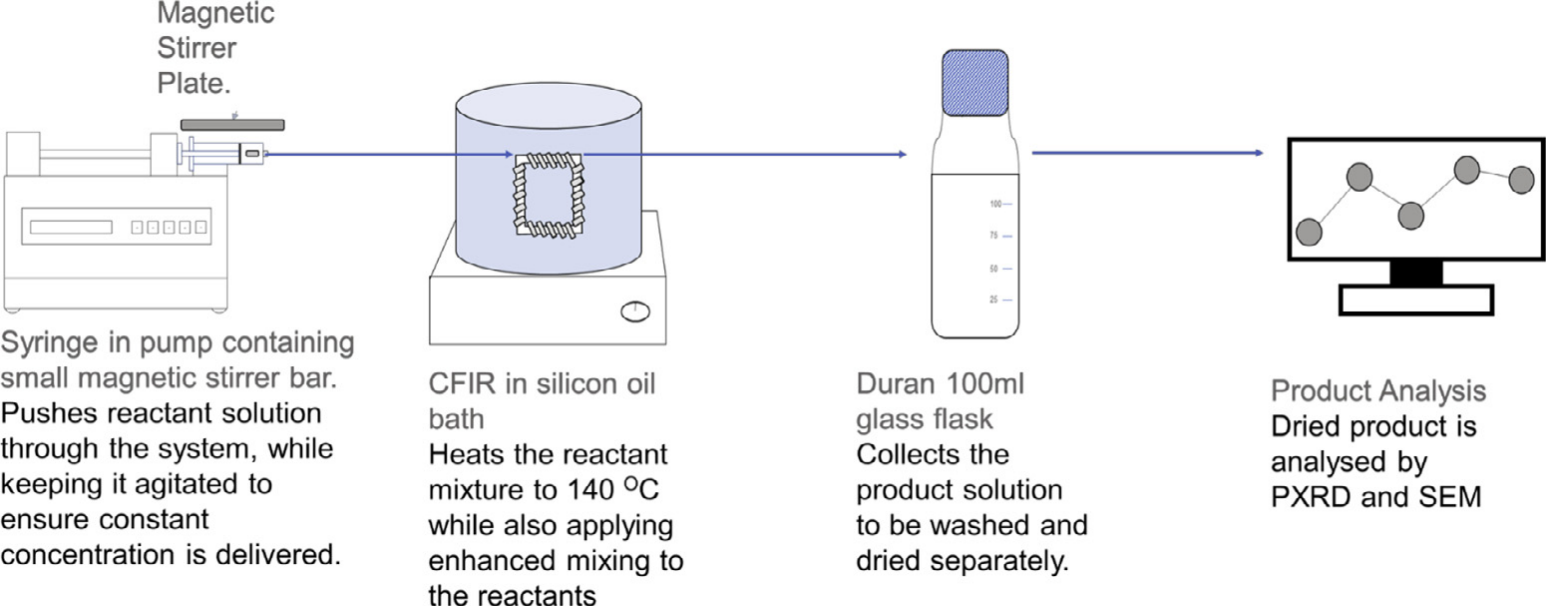
Schematic of protocol, showing the workflow from the syringe pump to the product.

Fig. 3 shows a schematic overview of the protocol. The CFIR is composed of polytetrafluoroethylene (PTFE) tubing (0.79 mm ID, 1/16” OD) coiled around 3D printed support fabricated with commercial high-temperature resin. The CFIR is placed into an oil bath, to be heated by a magnetic stirrer hotplate. The inlet to this reactor is connected to a syringe via IDEX flangeless fittings and threaded luer adapters (exact models provided in the Equipment section), with the syringe then sitting in the syringe pump once the reactant solution has been drawn up. This is then pumped through the heated system at a constant rate, with the product collected in a 100 ml glass flask at the end. This product then requires washing with fresh DMF and methanol and then drying to remove any solvent present in the materials pores. The UiO-67 is then analysed by PXRD and SEM.

## Materials

### Chemicals

Zirconium chloride (98%, Cat number: L14891) and silicon oil (Cat number: A12728.36) were purchased from Alfa Aesar. Benzoic acid (99%, Cat number: 237766) and biphenyl dicarboxylic acid (BPDC; 95%, Cat number: 091522) were purchased from Fluorochem. Methanol (>99.9%, Cat number: 34860-2.5L-R) and dimethylformamide (DMF; >99.9%, Cat number: 27054) were purchased from Sigma Aldrich. All the reagents were used as received without further purification.

### Equipment

• Syringe pumps (Fusion 101, Chemyx)
• Magnetic Stirrers (IKA, C-MAG HS7) with Electronic contact thermometer (IKA, ETS-D5, resolution of 0.1 °C)
• Tubing (PTFE, 1/32 ID 1/16 OD, Adtech)
• Centrifuge (Centurion 2000 Series)
• X-Ray Diffractor (Bruker D8,1.54 A, 2θ = 5^o^ - 45^o^)
• Flangeless Fitting (PEEK, 1/16” OD, IDEX Health & Science), Catalogue number: XP-283
• Luer Adapter (Female Luer x Female 1/4-28 Flat Bottom, IDEX Health & Science), Catalogue number: P-628
• Syringe (10 ml HSW Air Tite All Plastic)
• Scanning Electron Microscope (Carl Ziess EVO MA15)

## Equipment setup

### Coiled flow invertor reactor

The design of the CFIR requires a skeleton-based structure to be formed using AutoCAD software initially, so a 2D wireframe view was therefore selected as the preferred visual style to begin with. A typical procedure to draw the skeleton-based structure (helix diameter: 5 mm, pitch distance: 3 mm, and total length: about 3.16 m) involves:

1. A circle of radius 5 mm is first created. A helix, of height 75 mm, is then created with a base and top radius identical to the initial circle. The helix turn properties are altered to 25 turns with a turn height of 3 mm. The centre of the helix is then translated a distance 10 directly above the centre of the initial circle. This sequence of steps is illustrated below in Fig. 4.

**Fig. 4:**
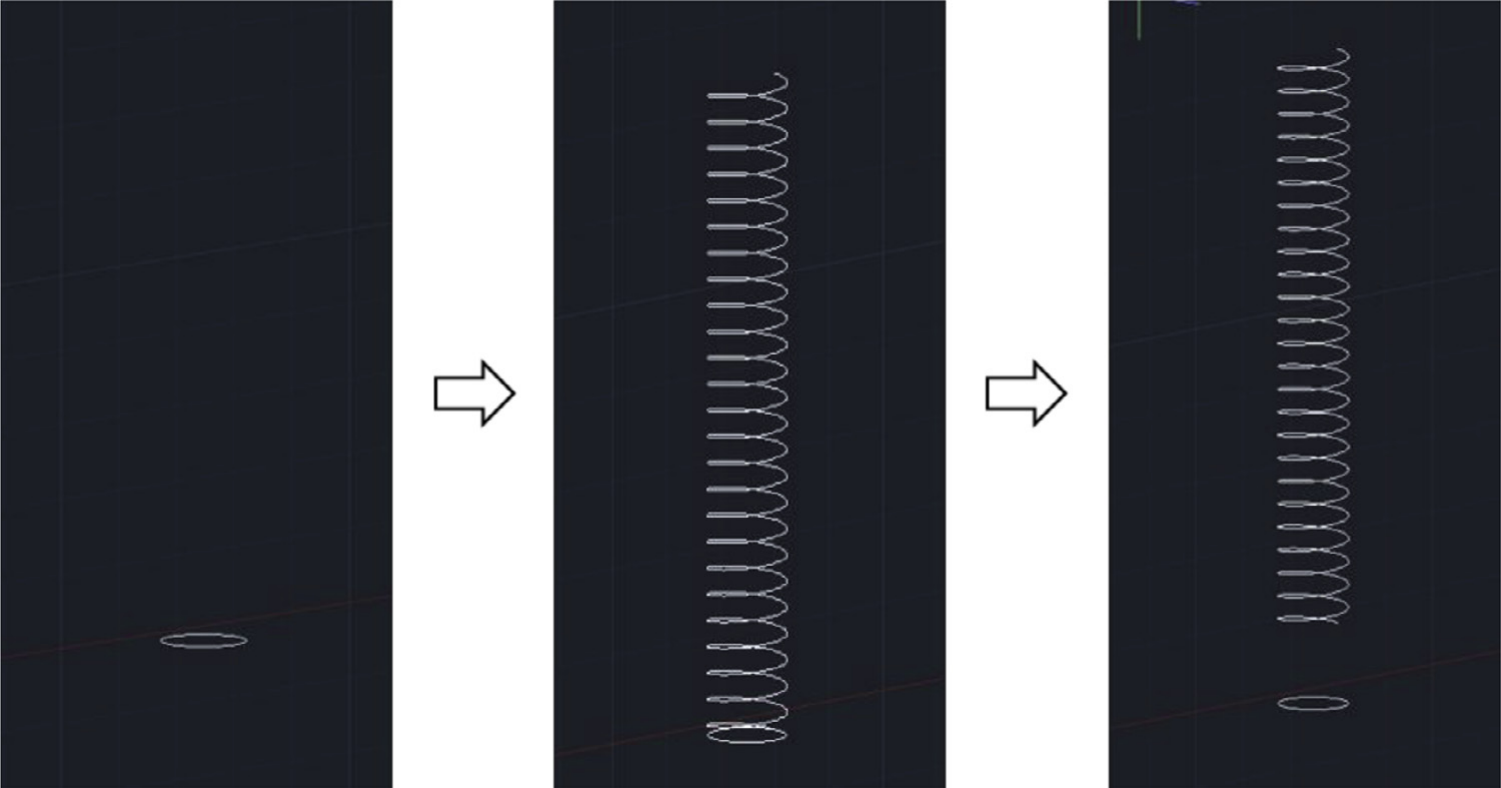
Series of steps used to form the basic helix shape in AutoCAD.
2. This assembly is copied, rotated horizontally, and aligned in a manner to ensure a continuous link is formed between the helixes and is repeated to complete the four flow inversions. It is important to ensure that the helixes meeting in one of the corners (in this case the top right) do not meet, as that will be where the inlet and outlet holes are placed. At this corner, a straight line is placed at the end of the helixes, which will be removed from the final structure to form the inlet and outlet channels, shown in Fig. 5. Also included in a support square of 2.5 mm x 2.5 mm x 10 mm that is placed adjacent to the inlet and outlet lines, to affixed at a later stage. This support square is where the inlet/outlet tubing will pass through and be held securely in the structure. Once these steps have been finished, the wireframe is complete, which will be used to form the final structure through extrusion.

**Fig. 5:**
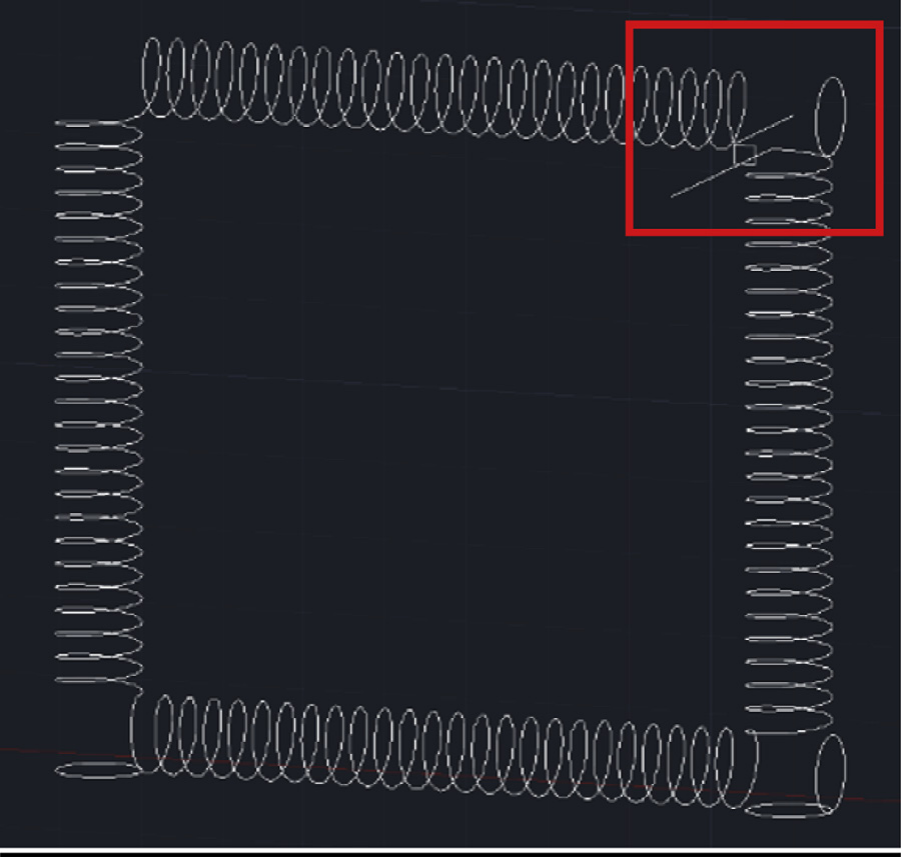
Wireframe CFIR structure, with the inlet/outlet corner highlighted.
3. To build the cylindrical supports, sweep paths need to be defined in the structure. To achieve this, firstly create a line of length 95 mm from the centre of each stand-alone circle through the centre of the helix. Then, using the ‘Sweep’ operator, select the circle as the object to sweep and then use the line drawn as the path for the sweep. Repeat this for each of the 4 circles/helixes. This sequence of steps is shown below in Fig. 6.

**Fig. 6:**
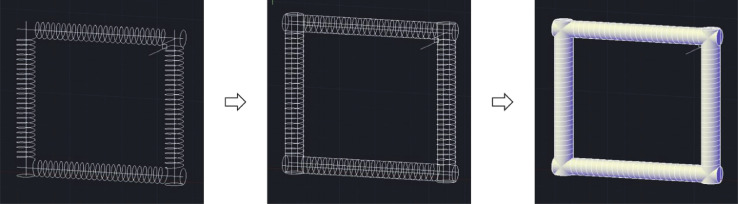
Sequence of AutoCAD steps used to sweep through the wireframe structure and form the 3D cylinders.
4. Draw a circle of radius 1.6 mm, then using the ‘Sweep’ operator again, sweep the circle through the structure using the helixes as a path. Once this has been done for each of the four helixes, subtract this structure from each cylinder using the ‘solid subtract’ function. This will leave a path for the tubing in the structure (Fig. 7).

**Fig. 7:**
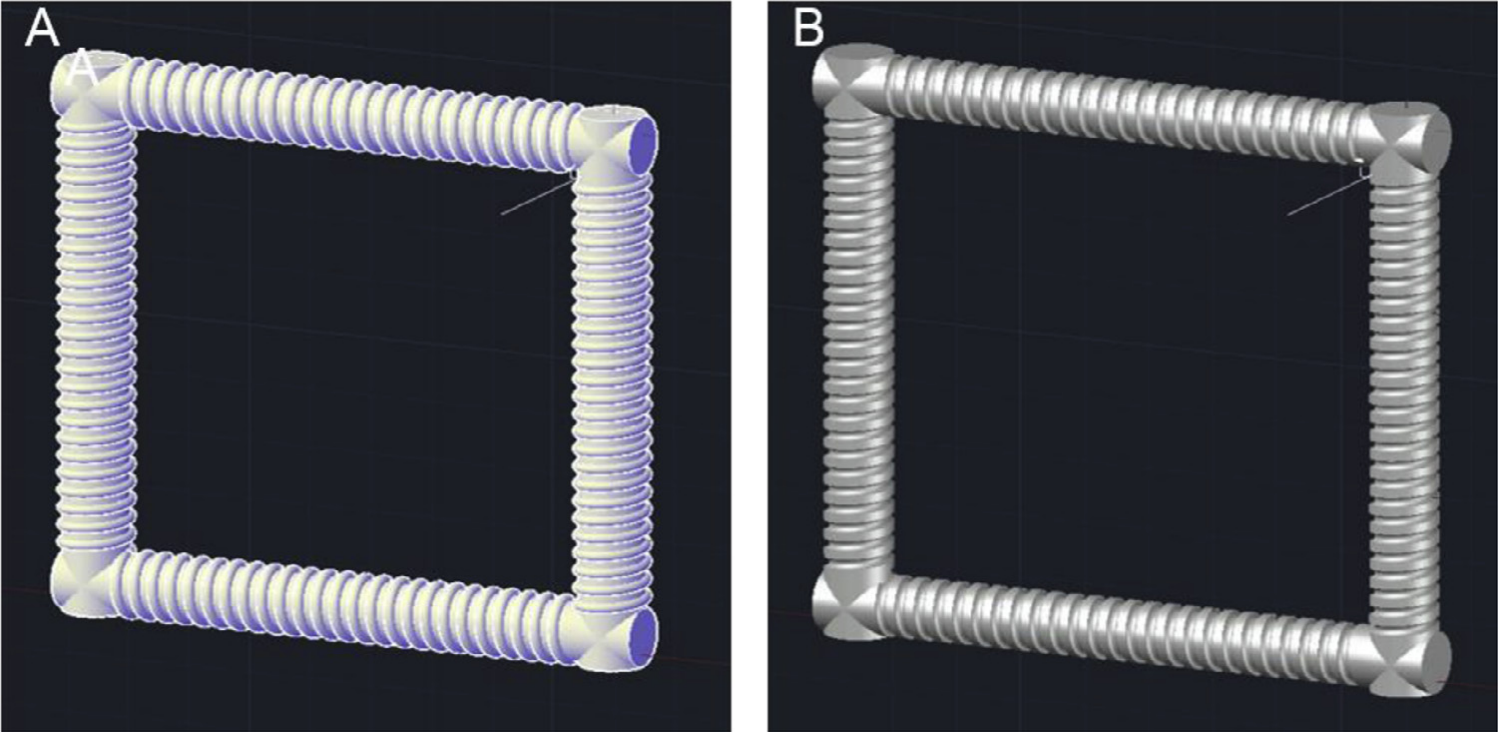
(A) structure with circle swept through the helix wireframe, and (B) structure with the channel formed through the subtraction of the helical sweep through.
5. Using this same method, the inlet and outlet channels can be formed. By sweeping through the 1.6 mm circle and subtracting the structure, it forms two holes in the piece, which will be used to secure the tubing. This is shown in Fig. 8.

**Fig. 8:**
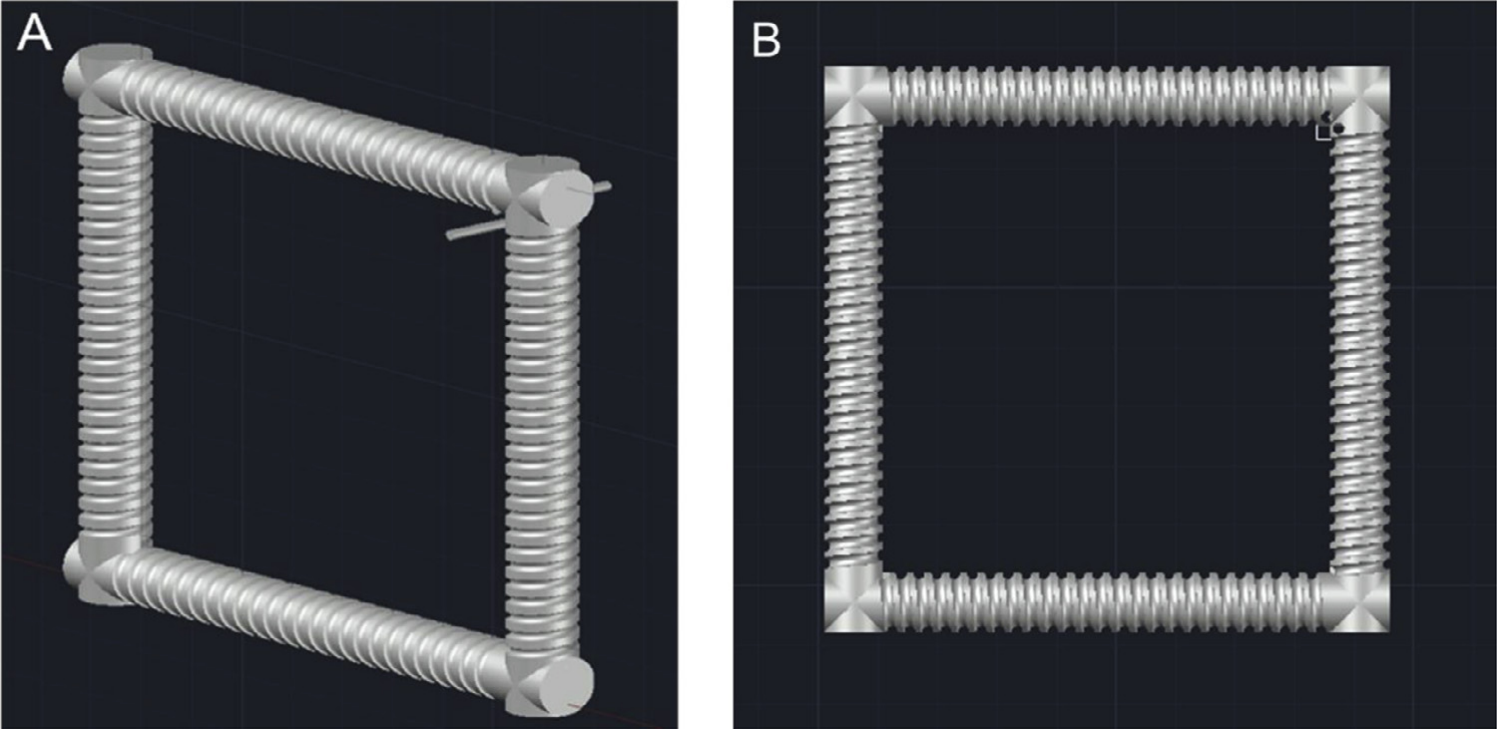
(A) Structure with inlet/outlet lines swept through by the 1.6 mm circle, (B) Structure with these sweep throughs subtracted.
6. To complete the square support at the inlet/outlet corner, a straight line of length 10 mm is draw through the centre of the square, so that a length of 5 mm is on each side of the square. Using this line as a guide, sweep the square and it will form the cuboid support. These 2 steps are shown in Fig. 9.

**Fig. 9:**
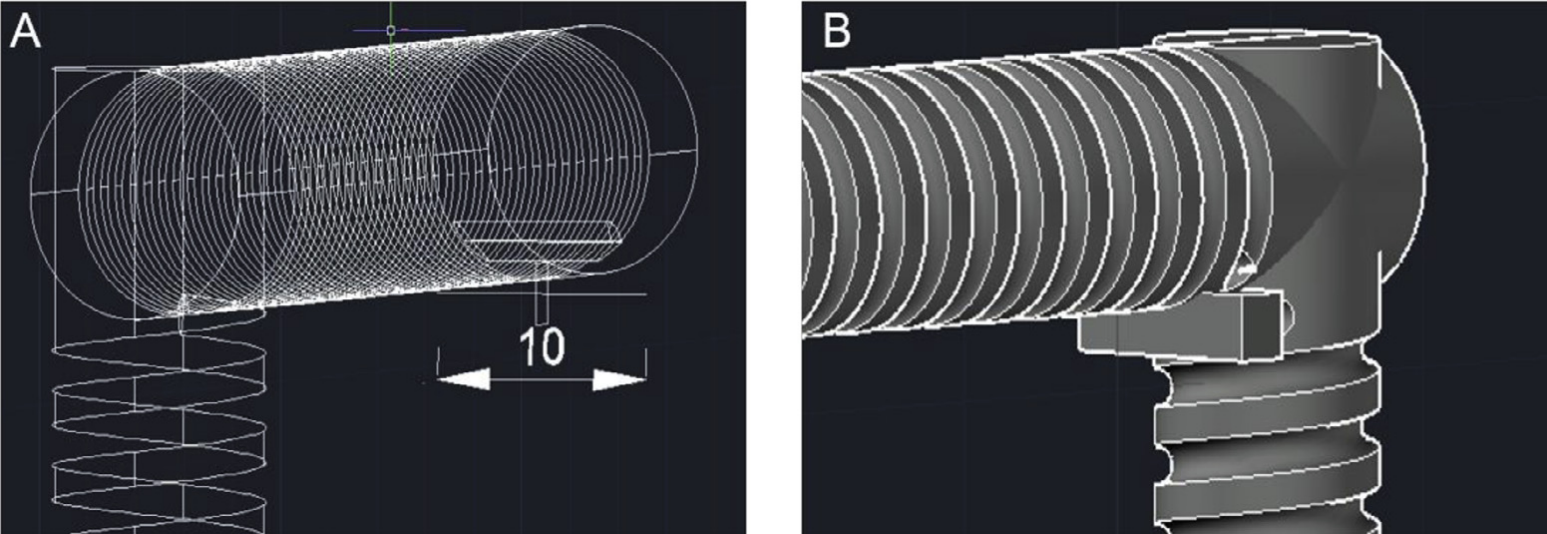
(A) Image highlighting the line drawn through the support square in the inlet/outlet corner, (B) Inlet/Outlet corner once the square has been swept through using this line.
7. Finally, to merge all the structures, first highlight all the pieces and use the ‘solid union’ function to combine them. This structure was saved as an STL file and then printed using Formlabs form 2 3D printer with high temp resin, followed by curing with UV light for 8 h.

### Microfluidic connections

**Fig. 10:**
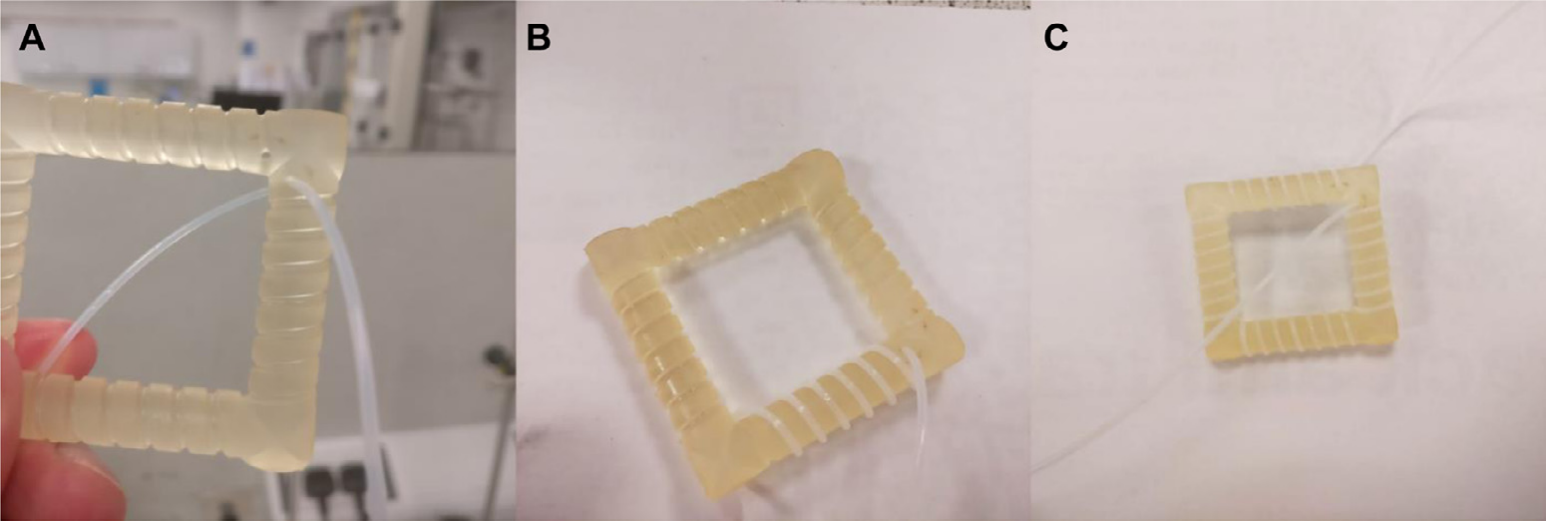
(A) The CFIR with tubing pressed through the corner inlet, (B) the CFIR with the tubing wrapped around 1 side, and (C) the fully completed CFIR with the tubing now pressed through the second corner hole.

Firstly, the tubing needs to be coiled around the CFIR unit. The easiest way to do this is to cut a length of tubing that will be slightly longer than the amount needed for the CFIR, to allow for inputs/outputs to be attached easily. In this case the amount needed for the CFIR was 3.146 m, so 3.5 m was cut to give about 18 cm excess for each side. The tubing is then pushed through one of the inlet holes in the corner (Fig. 10a), to then be wrapped fully around the CFIR, before being pushed through the other hole present in the corner. The tubing should be wrapped tightly around the CFIR, ensuring the length of tubing will be correct while also allowing for it the be moved easily.

**Fig. 11:**
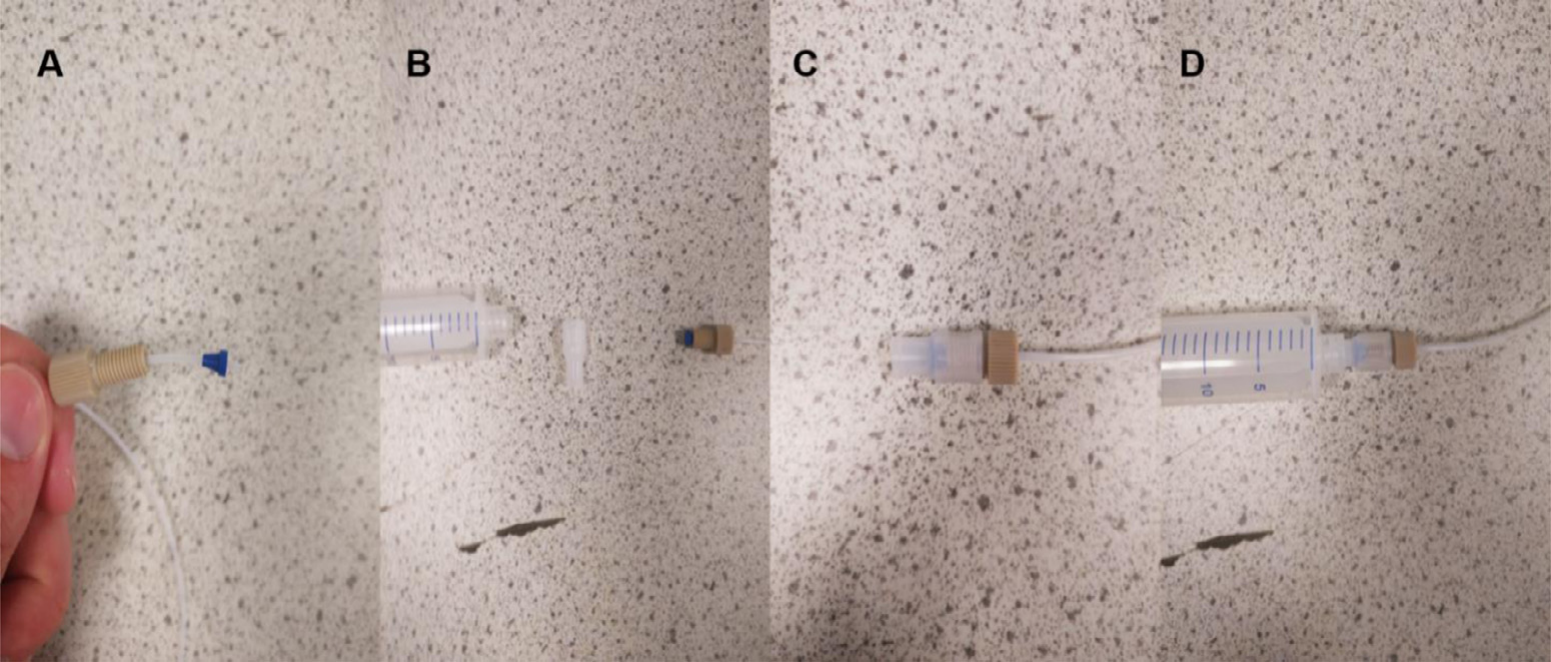
(A) Tubing with fitting and ferrule attached (B) Photo showing the syringe, luer adaptor and tubing with the ferrule now tightly attached. (C) Luer adaptor now screwed onto the end of the tubing. (D) Tubing now connected to the syringe.

The next step is to connect the inlet tubing to the syringes. The connecting ports are made of 3 parts: a ferrule, a flangeless fitting and a threaded luer adapter to the syringe (Fig. 11). Firstly, the tubing is inserted through the flangeless fitting, with the thread facing the inlet. The tubing is then also inserted through the ferrule, with the flat side facing into inlet. This is then tightly screwed into the threaded luer adapter, securing the tubing, and ensuring no leakage. The connector is then simply screwed onto the front of a syringe. This is shown in Fig. 11.

Once this has been set up, the CFIR unit can be placed in a Duran 900 ml crystallising dish with a stirrer bar placed inside, on top of the magnetic stirrer hotplate. Silicon oil will then be poured over this to cover the reactor, ensuring uniform heating. The outlet tubing will then be placed in a chosen collection vessel, which in this case was 100 ml Duran flasks.

### Slurry delivery system

**Fig. 12:**
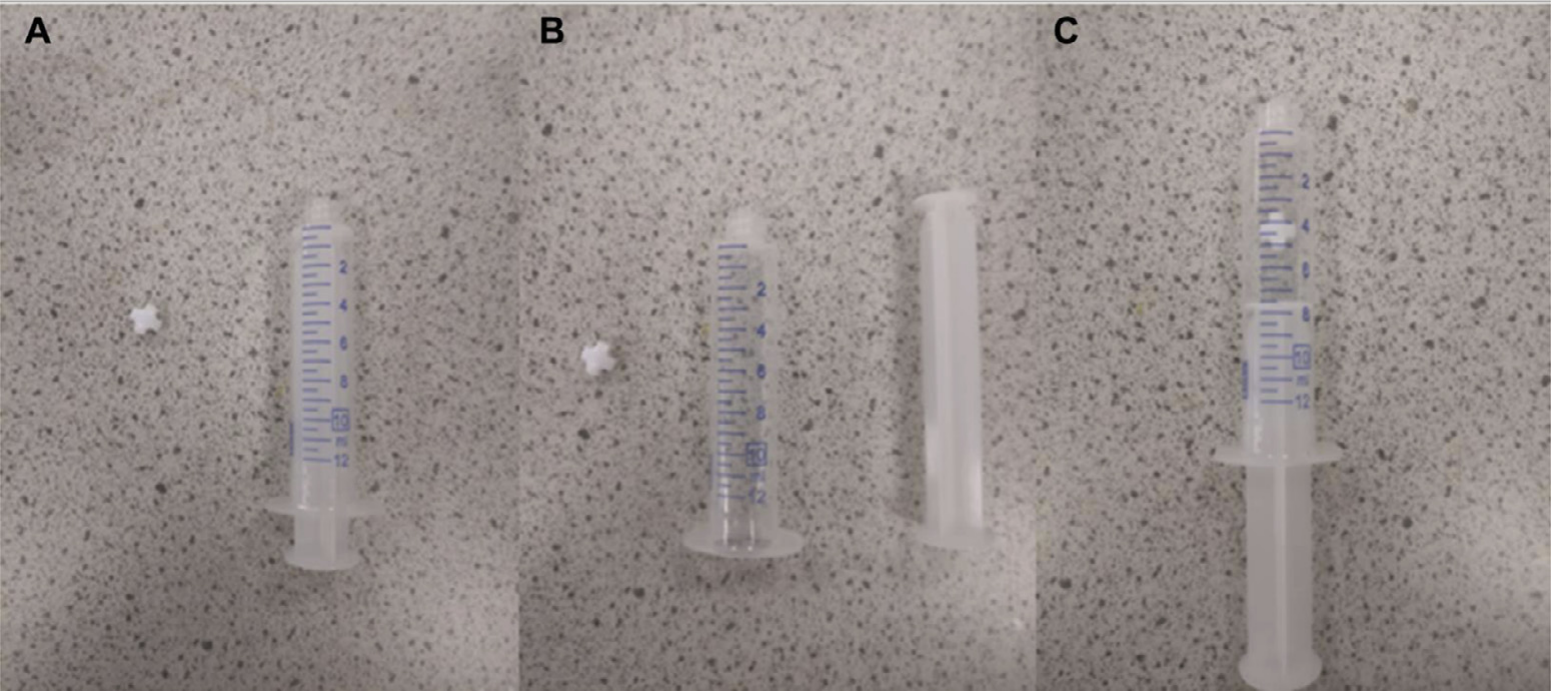
(A) Syringe and magnet next to each-other, (B) Image showing syringe with plunger removed, (C) Image showing syringe with magnet placed inside.

An issue found in this procedure is that the reactant mixture does not fully dissolve at room temperature, and so the suspension collapses over time in the syringe. While it may be well mixed after the sonication, the reactants will not be fully dissolved at room temperature and will crash out of solution over time. This means that the reactant concentration entering the system will be changing over time, giving variable reaction conditions over the course of the run. To ensure that the concentration entering the system is constant, the reactant solution within the syringe needs to be constantly mixed. This can be achieved by placing a very small magnetic stirrer bar inside the syringe and holding a small magnetic stirrer plate over the syringe pump while the reaction process is occurring. This section goes through the steps of setting this up. Firstly, as can be seen in Fig. 12A below, the plunger is pulled out of the syringe to allow the magnet to be placed inside (in this case a 5 mm bar), with the plunger then reinserted.

**Fig. 13:**
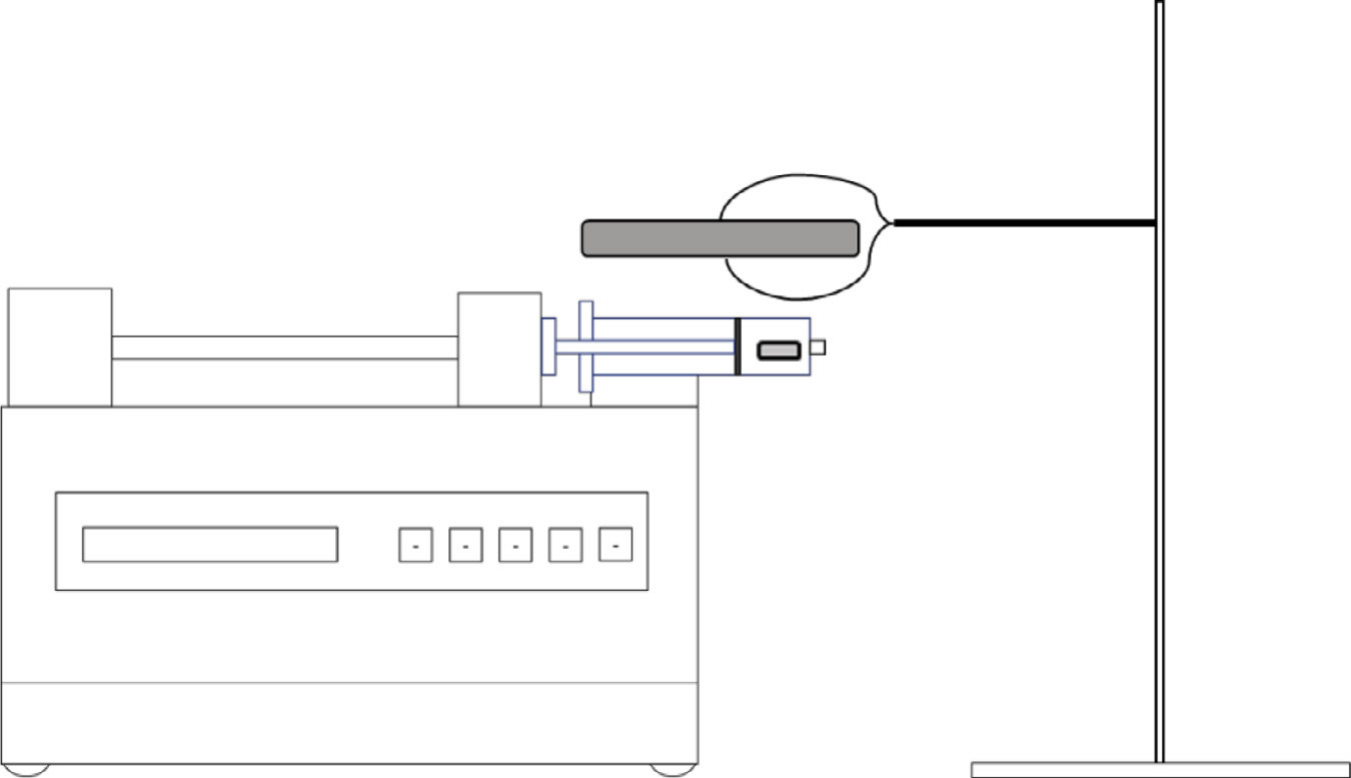
Scheme showing the general setup for the slurry delivery system, with the grey rectangle representing the magnetic stirrer plate and the smaller grey cuboid representing the magnetic stirrer bar.

Using this syringe, the reaction mixture is drawn up. Some air will be in the syringe when the solution is added, so this needs to be removed before connection to the system. Once it has been connected to the system, place the small stirrer plate above the syringe using a clamp stand. A simple scheme for this is shown in Fig. 13. Turn on the stirrer plate and set it to around 1500 RPM, where the bar will spin constantly in one position, with higher speeds causing the bar the move erratically around the syringe. Once this is setup, the pump can be started.

To recover the magnet at the end, fully empty the syringe of any leftover solution and then pull out the plunger. Wash the magnet with acetone and dry in an oven to be ready to use in the next run.

## Controlling the residence time

The residence time for this reaction will be controlled by the flowrate used with the syringe pump, which can be calculated by finding the volume of tubing present in the reactor. To quickly go through the calculations of the residence time in this experiment:

(1) $$t=\frac{V}{Q}=\frac{\frac{1}{4}\pi d^{2}L}{Q}$$

Where t is the residence time, *V* is the internal volume of the CFIR, d is the inner diameter of the tubing, L is the total length of the CFIR (only considering the part in the oil bath), and Q is volumetric flow rate. In this work, the inner diameter of the tubing is 0.79 ˣ 10^−3^ m, the total length of the reactor is 3.146 m, if the volumetric flow rate is set to be 0.052 ml/min, the residence time is then:

(2) $$t=\frac{V}{Q}=\frac{\frac{1}{4}\pi d^{2}L}{Q}=\frac{0.25\;\times \;3.14\;\times {\left(0.79\times 10^{-3}\right)}^{2}\times \;3.146}{0.052\;\times 10^{-3}}=30\;\text{min}$$

While the residence time may be 30 min, the overall time for the run will be much longer, as 10 ml of solution has to pass through the system at this rate, giving an overall run time of around 3 h. The residence time can be easily tuned by changing the volumetric flow rate.

## Experimental procedure

Firstly, the oil bath containing the CFIR was heated to 140 °C, which was chosen rather than 120 °C due to batch testing at 140 °C showing that increasing the temperature reduces the reaction time needed. ZrCl_4_ (0.26 mmol) and benzoic acid (1.28 mmol) were placed in a dry beaker before adding DMF (10 ml), adding a small amount of DMF at the start as a small amount of HCl gas will be released initially, due to the ZrCl_4_ reacting with any trace amounts of water present in the beaker/DMF added. This beaker was then sonicated for 1 min. Following this, BPDC (0.26 mmol) and distilled water (0.25 ml) was added to the solution, and then sonicated for a further 3 min. The use of water will increase the overall pH of the solution and so increase the deprotonation rate for the linker units, and so increase the rate of formation [19]. While all the reactants will be in the same syringe, this should not affect the result significantly as the reaction is extremely slow at room temperatures. A 10ml syringe with a small magnetic stirrer bar placed inside of it was then used to take up the reactant solution. Before attaching this syringe to the microreactor, 2 ml (which is greater than the total internal volume of the tubing of 1.72 ml) of fresh DMF was quickly pumped through the system, to ensure no previous reactants/products were present. The syringe with the reaction mixture was then attached to the microfluidic reactor and placed in the pump, with a small magnetic stirrer plate set up above the syringe. The pump was then started at a rate of 0.052 ml/min, which gave a residence time of 30 min. The reaction was performed for 2.5 h before the magnetic stirrer bar stops spinning, at which point the syringe was removed and a new syringe containing 5 ml of fresh DMF was attached. 2 ml of this fresh DMF was pushed through at the same flowrate, to ensure all reactants/products in the system were evacuated in the appropriate residence time. Finally, another 2ml of fresh DMF was pushed through at a flowrate of 1 ml/min, to ensure any settled product/reactant in the system were removed. No severe build-up/blockages have been observed over multiple runs, with the process being repeatable. The product was washed with fresh DMF (2 × 15 ml) and then left to soak in methanol (15 ml) for 72 h, changing the methanol every 24 h. The solvent was removed each time by centrifuge (6000 rpm, 20 min) and then left to dry overnight in an oven at 110 °C. The product was then collected and analysed by powder X-ray diffraction (PXRD) and scanning electron microscopy (SEM). The PXRD analysed the powder sample from 2θ = 5^o^ – 45^o^, scanning for 45 min with a scan interval of 0.033^o^ using Cu Kα radiation. The SEM was performed using a Carl Ziess EVO MA15, which imaged the particles after being sputter coated with 25 nm of iridium.

### Timing

• Synthesis run: 2.5 h
• Washing Process: 72 h
• Drying Process: 12 h

Currently, the production rate of this process is extremely low, at 0.81 mg h^−1^, but there are several ways in the future to improve this. The washing and drying times will be the same, independent of the amount of product present, so increasing the amount of product formed in the synthesis time would be vital. This could be achieved by increasing the size of the CFIR and the length of tubing it holds, allowing for higher flowrates while keeping the residence time constant. Multiple parallel reaction lines could be used as well, giving more product in the synthesis time. This process does show promise though, with a space time yield of 524 kg m^−3^ day^−1^, based on the volume of DMF used throughout the process. This is comparable to other continuous MOF production, with a previous plug flow reactor for UiO-66 showing a STY of 428 kg m^−3^ day^−1^ but can be improved upon by increasing the amount of product formed during the synthesis time [20].

## Anticipated results

### XRD patterns

**Fig. 14:**
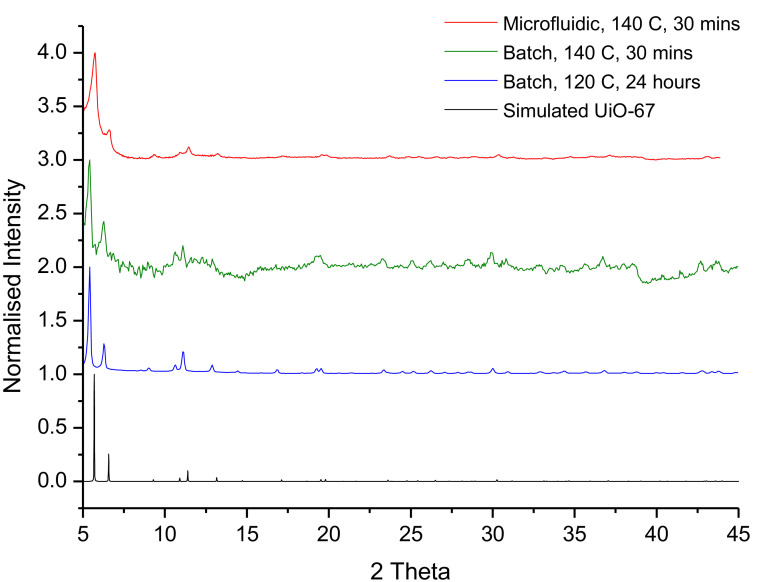
(Top to bottom) PXRD patterns for: Microfluidic synthesised UiO-67, Batch synthesised UiO-67 at 140 °C for 30 min, traditionally batch synthesised UiO-67 (24 h, 120 °C), simulated UiO-67 performed by Vesta. All patterns are over 2θ = 5 – 45^o^.

The final product show appears as a white powder, with a yield of ≈70 mg achieved in this experiment. Fig. 14 is the XRD pattern for the product, with comparison to the expected XRD patterns for a traditional batch UiO-67 product, a batch procedure performed under the same conditions as the CFIR reaction and a simulated spectrum performed in VESTA. All patterns show the key peaks at 2θ ≈ 5.70^o^ and 6.6^o^, confirming the product to be UiO-67, with the smaller peaks appearing also being characteristic of UiO-67. These peak positions are consistent with the simulated results shown and with previous work on UiO-67 [21]. The experimental peaks are slightly shifted to lower angles in the experimental batch results, potentially due to the sample not being completely dry at the time of analysis, with absorbed species from the air expanding the internal structure, leading to a decrease in angle.

Using the patterns shown, the Scherrer equation was applied, shown below in Eq. (3), to calculate the crystal sizes for the various MOF products.

(3) $$d=\frac{K\lambda }{\beta \text{cos}\theta }$$

Where d is the crystal size in Angstroms, K is the shape constant, which is between 0.8 and 1, usually assumed to be 0.9, λ is the wavelength of X-Rays used (1.542 Å), *β* is the Full-Width Half-Maximum (FWHM) of the peak being analysed, with the instrumental line broadening subtracted, inputted in Radians, and *θ* is the Bragg angle, also inputted in radians.

As this equation was applied to each synthesised product it gave a calculated crystal sizes of 177 nm, 40 nm, 28 nm for the traditionally synthesised batch UiO-67, the shorter batch process performed at 140 °C and the CFIR microfluidic product respectively. This smaller size is to be expected for the shorter reaction times and higher temperatures, increasing the rate of nucleation and the lack of time stopping the growth. This. should not be taken as the particle size, but to show the general trend in decreasing size, with SEM confirming the true size to be larger.

## SEM images

**Fig. 15:**
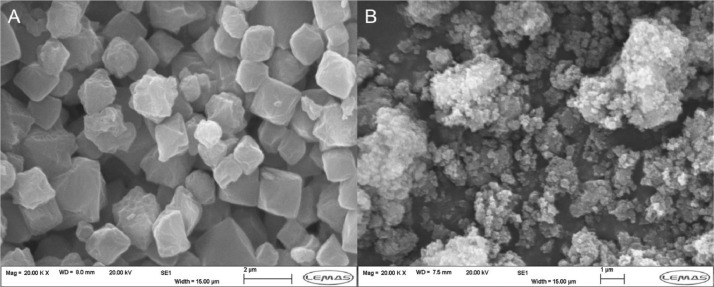
(A) SEM image for UiO-67 product formed through traditional 24 h 120 °C batch process, (B) SEM image for UiO-67 product formed through microfluidic CFIR process.

SEM images were taken for these three samples, which were prepared by sticking to a carbon tab on a SEM stub and then sputter coated with 25 nm of Iridium. Fig. 15 shows SEM images for the traditionally synthesised batch UiO-67 and the product obtained using the CFIR. The traditionally synthesised UiO-67 particles (Fig. 15a) show clearly defined edges, with the particles ranging in size from ~500 nm to over 3 µm in diameter and separate from each other. The particles formed in the CFIR (Fig. 15b) are much smaller and show a narrower distribution of sizes, ranging from ≈200 nm to ≈400 nm and appear as separate particles.

**Fig. 16:**
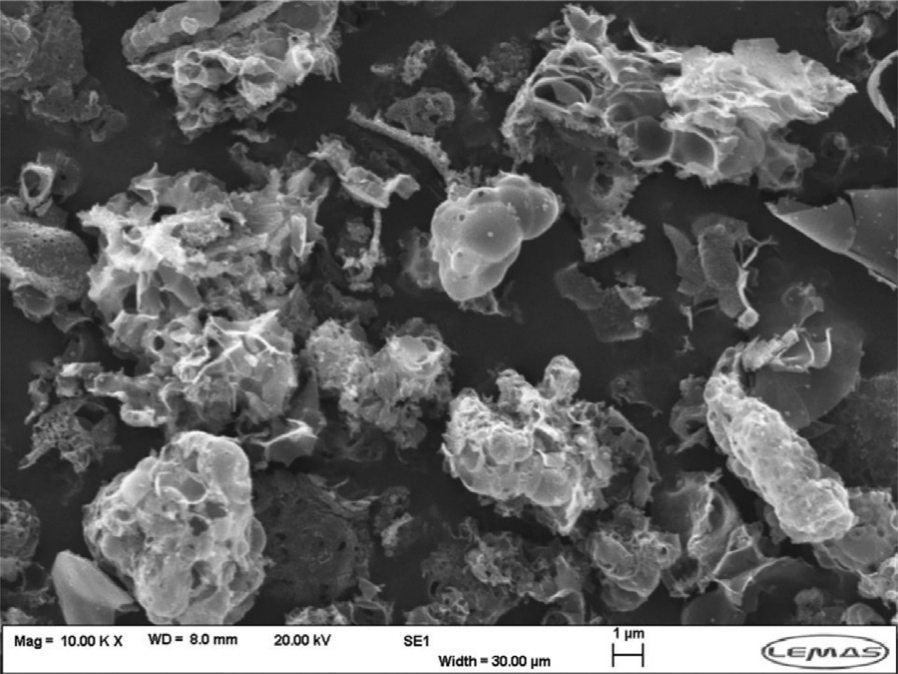
SEM image for UiO-67 product from 30 min 140 °C batch synthesis.

Fig. 16 shows the SEM image for the batch synthesised UiO-67 which used the same reaction conditions as the CFIR method (140 °C, 30 min, 50 eq H_2_O). As can be seen, the particles here are no longer distinct, with no clear edges present. This suggests that this product has a lower level of crystallinity than the products formed in the CFIR, with use of the CFIR has increasing the rate of reaction through enhanced mass/heat transfer, forming a high-quality crystalline product in a fraction of the time taken traditionally. It is also worth noting that the 140 °C batch run had to be scaled up considerably (by a factor of 6 when compared to the microfluidic synthesis) in order to form enough product (≈50 mg) for XRD and SEM analysis.

The characterisation results, incorporating both XRD and SEM, confirm that by using a CFIR microreactor crystalline UiO-67 can be produced, when similar conditions in batch do not. Furthermore, the particles produced using a CFIR are smaller and less varied in size than those produced via the typical batch conditions.

## References

[1] Hoskins B.F., Robson R. (1990). Design and construction of a new class of scaffolding-like materials comprising infinite polymeric frameworks of 3d-linked molecular rods. a reappraisal of the Zn(CN)2 and Cd(CN)2 structures and the synthesis and structure of the diamond-related framework. J. Am. Chem. Soc..

[2] Czaja A.U., Trukhan N., Müller U. (2009). Industrial applications of metal-organic frameworks. Chem. Soc. Rev..

[3] Chen D.T., Bi J.R., Wu J., Kumar A. (2020). Zirconium based nano metal–organic framework UiO-67-NH2 with high drug loading for controlled release of camptothecin. J. Inorg. Organomet. Polym. Mater..

[4] Bai Y., Dou Y., Xie L.H., Rutledge W., Li J.R., Zhou H.C. (2016). Zr-based metal-organic frameworks: Design, synthesis, structure, and applications. Chem. Soc. Rev..

[5] Bugaev A.L., Guda A.A., Lomachenko K.A., Kamyshova E.G., Soldatov M.A., Kaur G., Øien-ØDegaard S., Braglia L., Lazzarini A., Manzoli M., Bordiga S., Olsbye U., Lillerud K.P., Soldatov A.V., Lamberti C. (2018). Operando study of palladium nanoparticles inside UiO-67 MOF for catalytic hydrogenation of hydrocarbons. Faraday discussions.

[6] Yang Q., Wang Y., Wang J., Liu F., Hu N., Pei H., Yang W., Li Z., Suo Y., Wang J. (2018). High effective adsorption/removal of illegal food dyes from contaminated aqueous solution by Zr-MOFs (UiO-67). Food Chem.

[7] Barkhordarian A.A., Kepert C.J. (2017). Two new porous UiO-66-type zirconium frameworks; open aromatic N-donor sites and their post-synthetic methylation and metallation. J. Mater. Chem. A.

[8] Faustini M., Kim J., Jeong G.Y., Kim J.Y., Moon H.R., Ahn W.S., Kim D.P. (2013). Microfluidic approach toward continuous and ultrafast synthesis of metal-organic framework crystals and hetero structures in confined microdroplets. J. Am. Chem. Soc..

[9] Tai S., Zhang W., Zhang J., Luo G., Jia Y., Deng M., Ling Y. (2016). Facile preparation of UiO-66 nanoparticles with tunable sizes in a continuous flow microreactor and its application in drug delivery. Microporous Mesoporous Mater..

[10] Schaate A., Roy P., Godt A., Lippke J., Waltz F., Wiebcke M., Behrens P. (2011). Modulated synthesis of Zr-based metal-organic frameworks: from nano to single crystals. Chem. A Eur. J..

[11] Zhang R.Z., qing Huang Y., Zhang W., Yang J.M. (2018). Effect of particle size distribution of UiO-67 nano/microcrystals on the adsorption of organic dyes from aqueous solution. CrystEngComm.

[12] Hung L.H., Lee A.P. (2007). Microfluidic devices for the synthesis of nanoparticles and biomaterials. J. Med. Biol. Eng..

[13] Jahn A., Reiner J.E., Vreeland W.N., DeVoe D.L., Locascio L.E., Gaitan M. (2008). Preparation of nanoparticles by continuous-flow microfluidics. J. Nanoparticle Res..

[14] Uson L., Sebastian V., Arruebo M., Santamaria J. (2016). Continuous microfluidic synthesis and functionalization of gold nanorods. Chem. Eng. J..

[15] Sounart T.L., Safier P.A., Voigt J.A., Hoyt J., Tallant D.R., Matzke C.M., Michalske T.A. (2007). Spatially-resolved analysis of nanoparticle nucleation and growth in a microfluidic reactor. Lab. Chip.

[16] Kolmykov O., Commenge J.M., Alem H., Girot E., Mozet K., Medjahdi G., Schneider R. (2017). Microfluidic reactors for the size-controlled synthesis of ZIF-8 crystals in aqueous phase. Mater. Des..

[17] Wu K.J., Torrente-Murciano L. (2018). Continuous synthesis of tuneable sized silver nanoparticles: via a tandem seed-mediated method in coiled flow inverter reactors. React. Chem. Eng..

[18] Kurt S.K., Warnebold F., Nigam K.D.P., Kockmann N. (2017). Gas-liquid reaction and mass transfer in microstructured coiled flow inverter. Chem. Eng. Sci..

[19] Zahn G., Zerner P., Lippke J., Kempf F.L., Lilienthal S., Schröder C.A., Schneider A.M., Behrens P. (2014). Insight into the mechanism of modulated syntheses: In situ synchrotron diffraction studies on the formation of Zr-fumarate MOF. CrystEngComm.

[20] Rubio-Martinez M., Avci-Camur C., Thornton A.W., Imaz I., Maspoch D., Hill M.R. (2017). New synthetic routes towards MOF production at scale. Chem. Soc. Rev..

[21] Arrozi U.S.F., Wijaya H.W., Patah A., Permana Y. (2015). Efficient acetalization of benzaldehydes using UiO-66 and UiO-67: substrates accessibility or Lewis acidity of zirconium. Appl. Catal. A Gen..

